# Deletion of Tbk1 disrupts autophagy and reproduces behavioral and locomotor symptoms of FTD-ALS in mice

**DOI:** 10.18632/aging.101936

**Published:** 2019-04-30

**Authors:** Weisong Duan, Moran Guo, Le Yi, Jie Zhang, Yue Bi, Yakun Liu, Yuanyuan Li, Zhongyao Li, Yanqin Ma, Guisen Zhang, Yaling Liu, Xueqing Song, Chunyan Li

**Affiliations:** 1 Department of Neurology, The Second Hospital of Hebei Medical University, Shijiazhuang, Hebei 050000, People’s Republic of China; 2 Neurological Laboratory of Hebei Province, Shijiazhuang, Hebei 050000, People’s Republic of China; 3 Institute of Cardiocerebrovascular Disease, Shijiazhuang, Hebei 050000, People’s Republic of China; 4 Jiangsu Nhwa Pharmaceutical Co. Ltd, Nantong, Jiangsu 226000, People’s Republic of China

**Keywords:** Tbk1, frontotemporal dementia, amyotrophic lateral sclerosis, autophagy, p62

## Abstract

Haploinsufficiency of the protein kinase Tbk1 has shown to cause both amyotrophic lateral sclerosis (ALS) and frontotemporal dementia (FTD); however, the pathogenic mechanisms are unclear. Here we show that conditional neuronal deletion of Tbk1 in leads to cognitive and locomotor deficits in mice. Tbk1-NKO mice exhibited numerous neuropathological changes, including neurofibrillary tangles, abnormal dendrites, reduced dendritic spine density, and cortical synapse loss. The Purkinje cell layer of the cerebellum presented dendritic swelling, abnormally shaped astrocytes, and p62- and ubiquitin-positive aggregates, suggesting impaired autophagy. Inhibition of autophagic flux with bafilomycin A increased total Tkb1 levels in motor neuron-like cells *in vitro*, suggesting autophagy-dependent degradation of Tbk1. Although Tbk1 over-expression did not affect mutant SOD1 levels in SOD1^G93A^-transfected cells, it increased the soluble/insoluble ratio and reduced the number and size of SOD1^G93A^ aggregates. Finally, *in vivo* experiments showed that Tkb1 expression was reduced in SOD1^G93A^ ALS transgenic mice, which showed decreased p62 protein aggregation and extended survival after ICV injection of adeno-associated viral vectors encoding Tbk1. These data shed light on the neuropathological changes that result from Tbk1 deficiency and hint at impaired autophagy as a contributing factor to the cognitive and locomotor deficits that characterize FTD-ALS in patients with Tkb1 haploinsufficiency.

## INTRODUCTION

Amyotrophic lateral sclerosis (ALS) is a fatal neurodegenerative disease characterized by progressive loss of motor neurons in the cortex, brain stem, and spinal cord, although behavioral and cognitive symptoms often coexist with ALS [1–2]. TANK-binding kinase1 (Tbk1) is a multifunctional kinase involved in the regulation of various cellular processes, including immune response, inflammation, autophagy, cell proliferation, and insulin signaling [3]. Haploinsufficiency of Tbk1, resulting from heterozygous loss-of-function mutations causing ~50% reduction in Tbk1 levels, has been recently identified as a cause for ALS and frontotemporal dementia (FTD) [4–8]. FTD comprises a group of early-onset neurodegenerative syndromes that represent the most common form of dementia in people under age 60 [4–6]. Clinical signs and symptoms of ALS and FTD often overlap, implying a common etiology. About ~15% of FTP patients develop motor neuron deficits that are clinically consistent with ALS; conversely, between 10%-30% of ALS patients show signs of FTD [7–8]. However, the mechanism(s) by which Tkb1 deficiency promotes neurodegeneration and clinical manifestations of ALS-FTD is still unclear. Tbk1 germline knockout (KO) mice have been established, but these animals die embryonically at E14.5 due to liver degeneration [9]. The recent generation of Tbk1 conditional KO mice has provided an *in vivo* model for the study of Tbk1 function in immune cells [10–12]. However, the potential contribution of neuron-specific Tbk1 to ALS/FTD onset and progression remains to be determined.

Cu/Zn superoxide dismutase-1 (SOD1) mutations account for ~20% of familial ALS (fALS) forms [1, 13]. Transgenic mice expressing mutant SOD1 proteins such as G37R, G85R, and G93A show degeneration of motor neurons that mimics the clinical presentations and pathology of ALS [14–16]. SOD1 mutations associated with ALS generate gain-of-function mutants where pathological cellular effects, such as oxidative stress, mitochondrial dysfunction, endoplasmic reticulum stress, and inefficient protein degradation correlate with neuronal death [17–18]. Accumulating evidence suggests that mutant SOD1 can directly bind to functionally important proteins in neuronal cells, such as sequestosome 1 (p62) and voltage-dependent anion channel 1 (VDAC1), thereby reducing the physiologic function of these proteins by forming insoluble aggregates [19–20]. In the present work we evaluated the neuropathological, behavioral, and locomotor changes induced by Tkb1 deficiency in transgenic mice, the reciprocal impact of mutant SOD1 and Tkb1 deletion/overexpression in motor neurons, and their involvement in the autophagy pathway.

## RESULTS

### Conditional neuron-specific Tbk1 knockout leads to cognitive and motor dysfunction

**Figure 1:**
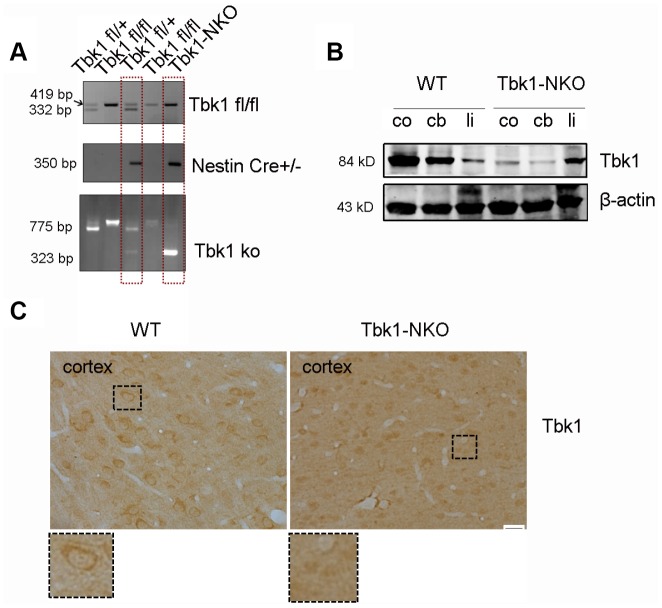
Conditional Tbk1-NKO mouse generation and genotyping. (**A**) Tbk1-NKO mice were established by crossing Tbk1^fl/fl^ mice with Nestin-cre mice, and genotyped by PCR. (**B**) Western blot expression of Tbk1 in the cortex (co), cerebellum (cb), and liver (li). (**C**) Tbk1 immunohistochemistry in brain cortex. Bar = 20 μm.

To investigate the role of Tbk1 in the central nervous system (CNS), we generated Tbk1 neuronal progenitor cell-conditional KO mice by crossing Tbk1-flox mice [10] with Nestin-Cre mice. The resulting Tbk1^fl/fl^Nestin-Cre (hereafter called Tbk1-NKO) mice, and Tbk1^+/+^Nestin-Cre wild-type (WT) control mice were genotyped by PCR (Figure 1A). Western blot analyses readily detected Tbk1 in the cortex, cerebellum, and liver of WT mice. In contrast, Tbk1 expression was normal in the liver, but barely detectable in the cortex and cerebellum of Tbk1-NKO mice (Figure 1B). Neuron-specific deletion was further confirmed through Tbk1 immunostaining (Figure 1C).

**Figure 2:**
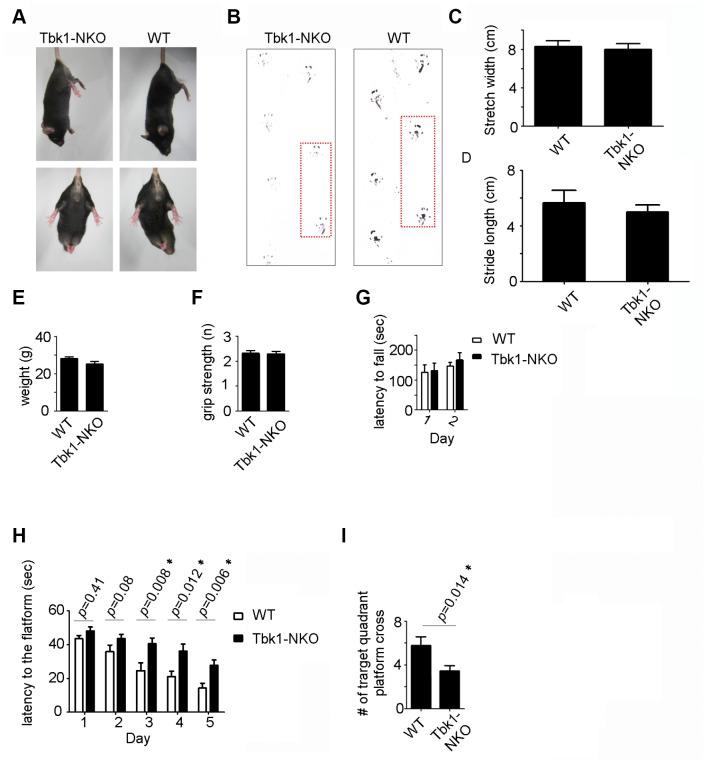
Behavioral evaluation of 5-month-old Tbk1-NKO mice. (**A**–**B**) Clasping and footprint assessment. (**C**–**D**) Stretch width and stride length measurements (n = 5). (**E**–**G**) Body weight, grip power, and rotarod latency (n = 13-21). (**H**–**I**) Morris water maze’s learning and memory test. Latency to reach the platform and number of target quadrant crosses (n = 13-21). *P < 0.05, compared to WT control.

**Figure 3:**
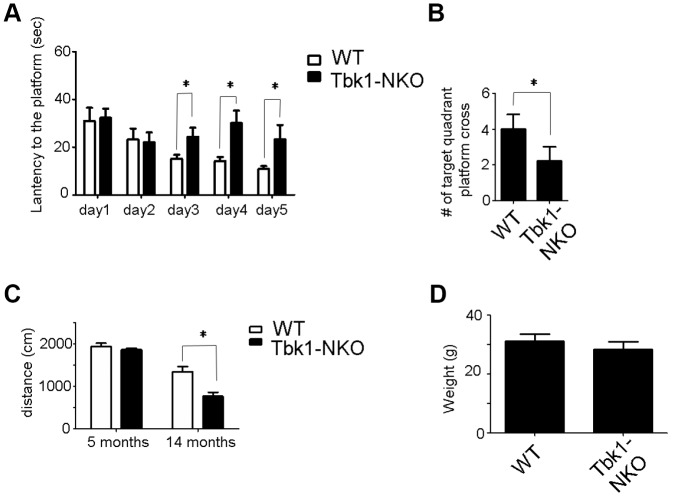
Behavioral evaluation of 14-month-old Tbk1-NKO mice. (**A**–**B**) Latency to reach the platform and number of target quadrant crosses in the Morris water maze (n = 9-10). *P < 0.05, compared to WT control. (**C**–**D**) Swimming distances and body weight (n = 9-10). *P < 0.05, compared to WT mice.

To assess the behavioral impact of Tbk1 deletion, locomotor and memory functions were evaluated in age-matched Tbk1-NKO and WT mice. Five-months-old Tbk1-NKO mice showed normal clasping and gait (footprint tracing) (Figure 2A–2D). Body weight, grip force, and latency to fall (Rotarod test) were also comparable in Tbk1-NKO and control mice (Figure 2E–2G). The Morris Water Maze test was next used to evaluate spatial learning and reference memory. Tbk1-NKO mice showed a significant increase in the time to reach the platform from day 3 to day 5, compared to WT mice. On day 6, a 60-second probe trial was administered in which the platform was removed. Trial results showed that the number of target quadrant crosses was reduced by 40% in Tbk1-NKO mice (Figure 2H, 2I; Supplementary Figure 1A). Interestingly, when the experiment was repeated in older mice (14 months), no significant decline in the time to reach the platform was observed over 5 days of training in Tbk1-NKO mice. However, swimming distances and successful target quadrant crosses were still reduced by 43.2% and 45%, respectively, compared to WT mice. Meanwhile, body weights were comparable between Tbk1-NKO and WT mice (Figure 3A–3D). These data showed that neuronal conditional Tbk1 knockout is associated with cognitive impairment and reduced locomotor activity in mice.

### Neuron-specific Tbk1 deletion induces morphological and biochemical alterations in neurons and glia

**Figure 4:**
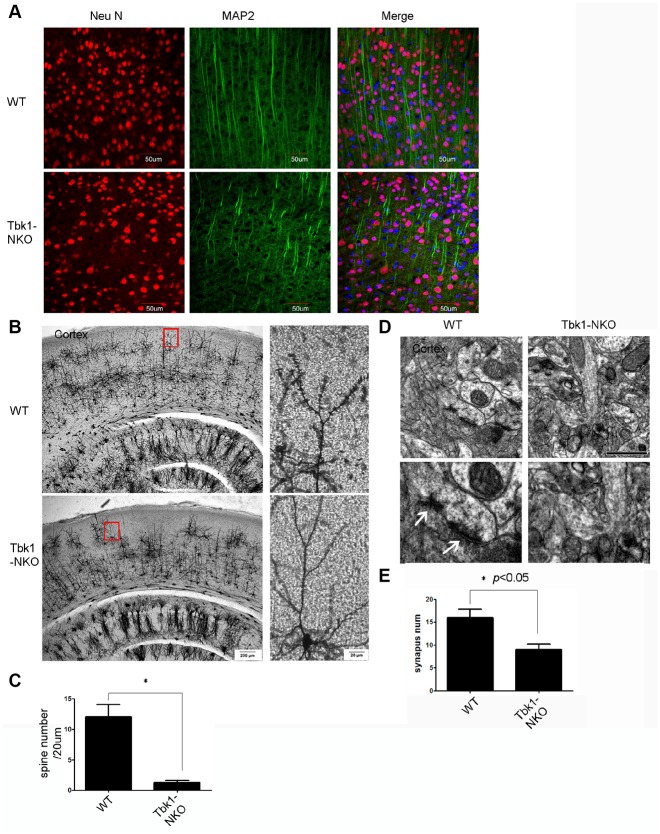
Dendro-synaptic alterations induced by Tkb1 deficiency. (**A**) NF and MAP2 immunofluorescence was used to visualize dendrites. (**B**) Golgi-Cox brain staining in Tbk1-NKO and WT mice (n = 3). (**C**) Analysis of dendritic spine density (n = 10). *P < 0.05, compared to WT mice. (**D**–**E**) Cortical synapse numbers, quantified from electron microscopy images (n = 6-7). *P < 0.05, compared to WT. Synapses are indicated by white arrows. Bar = 1 μm.

We applied both immunostaining and special stains to evaluate the effects of Tbk1 deletion on neuronal and glial morphology. Abnormally shaped neurons were observed in the cortex of Tbk1-NKO mice upon immunostaining against neurofilament (NF) and microtubule associated protein 2 (MAP2) as a dendritic marker (Figure 4A; Supplementary Figure 1C). Additionally, Golgi-Cox staining revealed a marked decrease in the density of dendritic spines in the cortex and hippocampus of Tbk1-NKO mice (Figure 4B, 4C; Supplementary Figure 2). Consistent with reduced dendritic spine density, TEM images showed that synapse numbers were also dramatic decreased in the cortex of Tbk1-NKO mice (Figure 4D, 4E).

**Figure 5:**
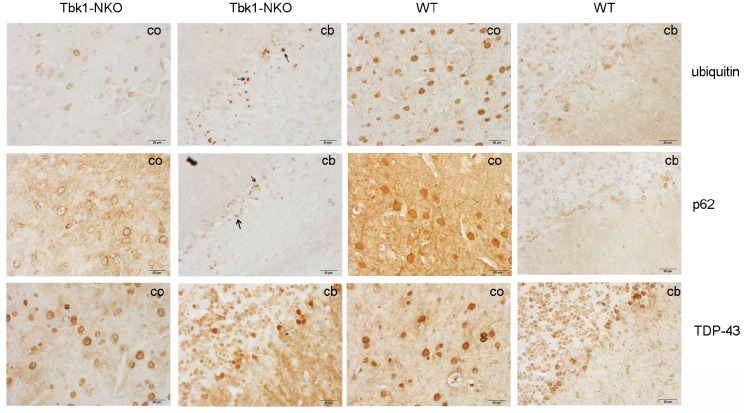
Identification of protein aggregates. Ubiquitin, p62, and TDP-43 immunostaining of the cortex (co) and cerebellum (cb) of Tbk1-NKO and WT mice (n = 3). Bar = 20 μm. Arrows indicate protein aggregates.

In view of the impaired behavioral performance of Tbk1-NKO mice, and based on evidence linking alterations in autophagy receptors such as p62 and neurodegenerative diseases -including ALS-, the expression of the autophagy-related proteins p62 and ubiquitin was evaluated by immunostaining in 14-month-old Tbk1-NKO mice. Whereas p62- and ubiquitin-positive aggregates were identified in the cerebellum, we did not observe TDP-43-positive inclusions, another common marker of ALS, FTD, and other neurodegenerative diseases (Figure 5).

**Figure 6:**
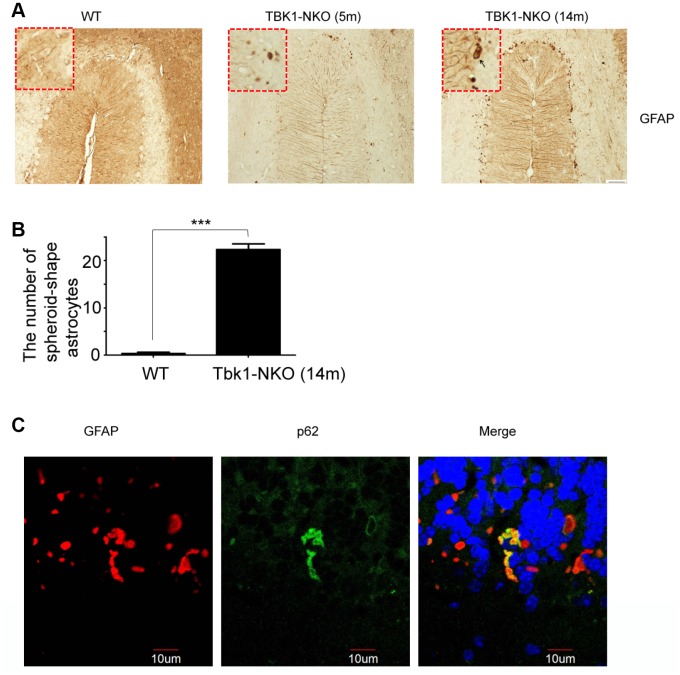
Astroglial alterations induced by Tkb1 deficiency. (**A**) GFAP immunostaining of the cerebellum of 5 and 14 months old mice (n = 3). Bar = 50 μm. Spheroidal astrocytes are indicated by arrows. (**B**) Quantification of astrocyte degeneration (n = 9); ***P < 0.001, compared to WT mice. (**C**) Double GFAP and p62 immunofluorescence of the cerebellum. Bar = 10 μm.

Astrocyte activation and degeneration have been reported in ALS. To examine possible pathologic changes in glia resulting from Tbk1 deficiency, microglia and astrocytes were analyzed, respectively, by IBA1 and GFAP immunostaining. While these analyses did not suggest obvious glial activation in the hippocampus or cortex (Supplementary Figure 1B), a stronger GFAP staining was observed, compared to WT mice and especially at 14 months of age, in the Purkinje cell layer of the cerebellum of Tbk1-NKO mice (Figure 6A, 6B). Furthermore, the presence of glial degeneration was suggested by a significant number of GFAP-positive astrocytes that presented an abnormal, spheroidal shape.

**Figure 7:**
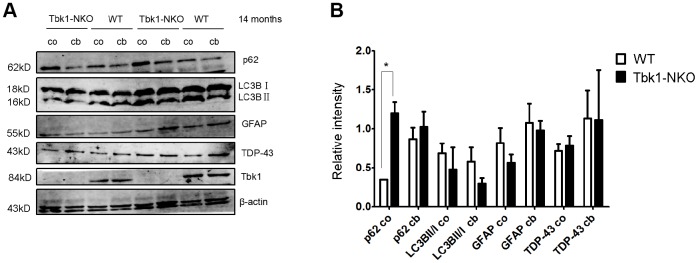
**Analysis of autophagy markers.** (**A**–**B**) Western blot expression analysis of p62, LC3B, GFAP, TDP-43, and Tbk1 in the cortex (co) and cerebellum (cb) of Tbk1-NKO and WT mice (n = 3); *P < 0.05, compared to control mice.

p62 (sequestosome 1) functions as a cargo receptor for autophagy and a shuttling factor of polyubiquitinated proteins to the proteasome [21]. p62-positive inclusions thought to result from impaired autophagy flux in neurons and astrocytes have been reported in specimens from ALS-FTD patients [22]. Using immunostaining, we detected formation of p62 aggregates in abnormal cerebellar astrocytes from Tbk1-NKO mice (Figure 6C). Western blots also showed elevated expression of p62 in both cerebellum and cortex of Tbk1-NKO mice, but the increase was significant only in the cortex (Figure 7A). The expression of other common autophagy markers, namely LC3B proteins, as well as GFAP and TDP-43 levels, were also studied by western blot. The LC3BII/LC3BI expression ratio was not changed in Tbk1-NKO mice compared to WT. Likewise, no differences between genotypes were seen for the expression of GFAP and TDP-43 (Figure 7A, 7B).

**Figure 8:**
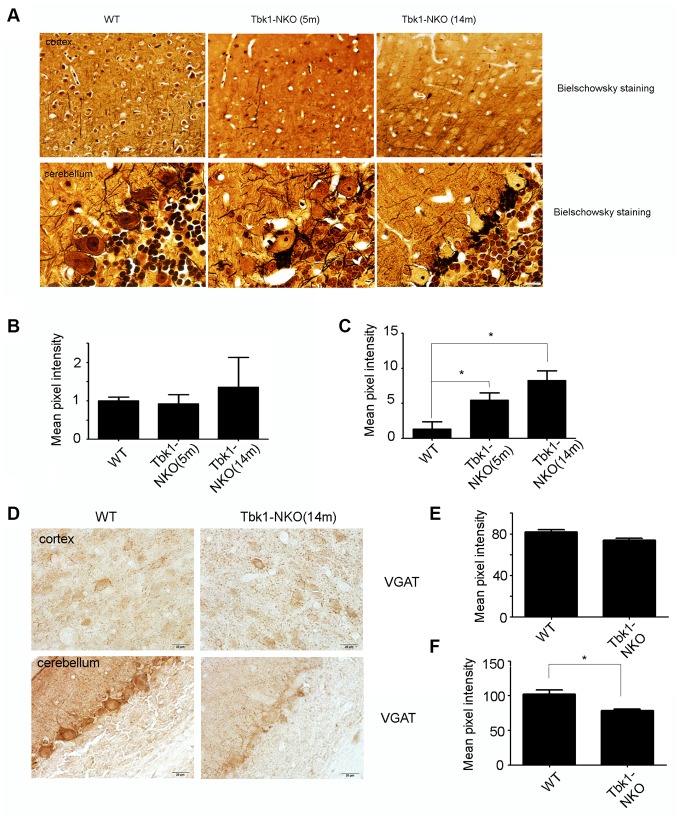
**Assessment of neuropathological changes.** (**A**) Bielschowsky staining of the cortex and cerebellum in mice aged 5 and 14 months (n = 3). A neurofibrillary tangle is indicated by a white arrow. Bar = 10 mm. (**B**–**C**) Quantification of dendritic densities (n = 3); *P < 0.05, compared to WT mice. (**D**) VGAT immunostaining of the cortex and cerebellum of mice at 5 and 14 months (n = 3). Bar = 10 mm. (**E**–**F**) Quantification of VGAT expression. Mean pixel intensity was analyzed using image J software (n = 3); *P < 0.05, compared to WT mice

To examine in greater detail the pathologic changes seen in the cerebellum and cortex of Tbk1-NKO mice, Bielschowsky staining was carried out in samples from 5 and 14 months old mice. Dendritic swelling in the Purkinje cell layer and neurofibrillary tangles in the cortex were evident in Tbk1-NKO mice aged 14 months (Figure 8A–8C). We speculated that the dendritic swelling observed in Purkinje neurons (and also possibly in basket and stellate cells that synapse with them) might be related to impaired GABA release. Supporting this hypothesis, reduced expression of the GABA transporter VGAT was observed by immunohistochemistry in the cerebellum of 14-month-old Tbk1-NKO mice (Figure 8D–8F).

### Tbk1 knockout impairs autophagy in motor neuron-like cells

**Figure 9:**
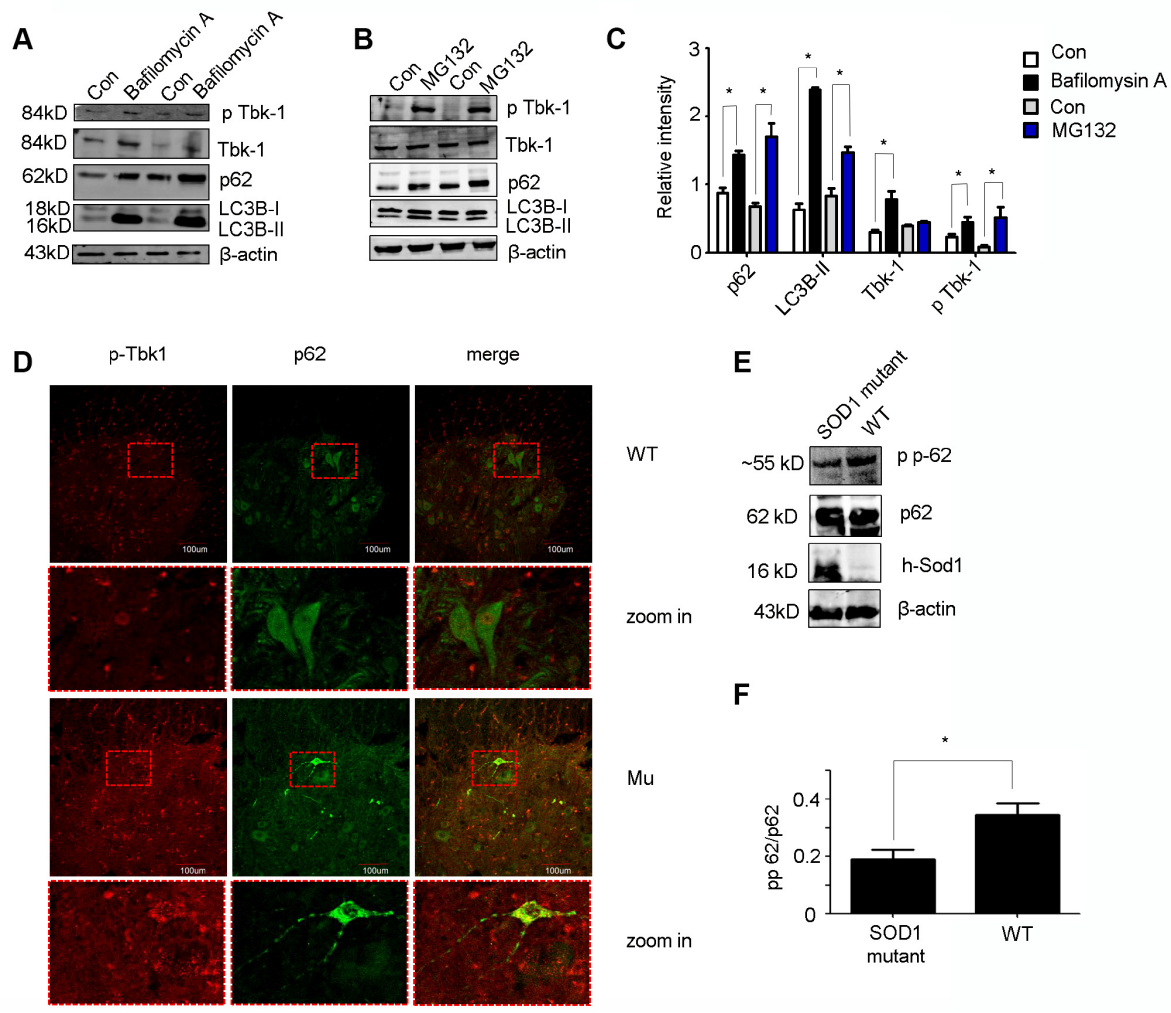
***In vitro* and *in vivo* protein expression analyses.** (**A**–**C**) Western blot analysis of p-Tbk1, total Tbk1, LC3B-II, and p62 in NSC-34 cells treated with bafilomycin A, MG132, or solvent control (Con) (n = 3); *P < 0.05, compared with Con. (**D**) Double immunofluorescence of p-Tbk1 and p62 in the lumbar spinal cord of SOD1^G93A^ mice and WT littermates. Bar = 100 μm. (**E**–**F**) Western blot analysis of p62 phosphorylation status in SOD1 mutant mice and WT controls (n = 3); *P < 0.05, compared to WT mice.

Previous studies demonstrated that upon phosphorylation by Tbk1, p62 efficiently binds cargo proteins and brings them to LC3B to promote autophagosome formation [23]. While there is evidence for proteasomal degradation of Tbk1 [24], it is unclear whether it can be degraded via autophagy. To address this question, we exposed motor neuron-like cells (NSC34) to the autophagy inhibitor bafilomycin A (20 nM; 6 h) or the proteasome inhibitor MG132 (1 μm; 24 h). As expected, the lysosomal inhibitor bafilomycin A increased the expression of both LC3B-II and p62. In parallel, an increase in total Tbk1 and, to a lesser degree, p-Tbk1 levels was also detected using western blot (Figure 9A–9C). Meanwhile. proteasome inhibition with MG132 markedly increased p-Tbk1 and p62 expression, induced only a moderate increase in LC3B-II, and did not affect total Tbk1 levels (Figure 9B, 9C). These data suggest that Tbk1 can be degraded via autophagic processes in neuronal cells.

Next, we examined whether p-Tbk1 was involved in p62 aggregation in motor neurons. Indeed, p-Tbk1 co-localized with p62 in the aggregates (Figure 9D). Mutant SOD1 is known to interact with p62 to form aggregates with disruptive effects on the protein degradation pathway [19, 25], which raises the possibility that mutant SOD1 could disrupt the ability of Tbk1 to regulate p62 function. To test this idea, the phosphorylation of p62 at Ser403 was assessed in cells transfected with a mutant SOD1 protein (SOD1^G93A^). We found that SOD1^G93A^ expression partially decreased p62 phosphorylation (Figure 9E, 9F).

**Figure 10:**
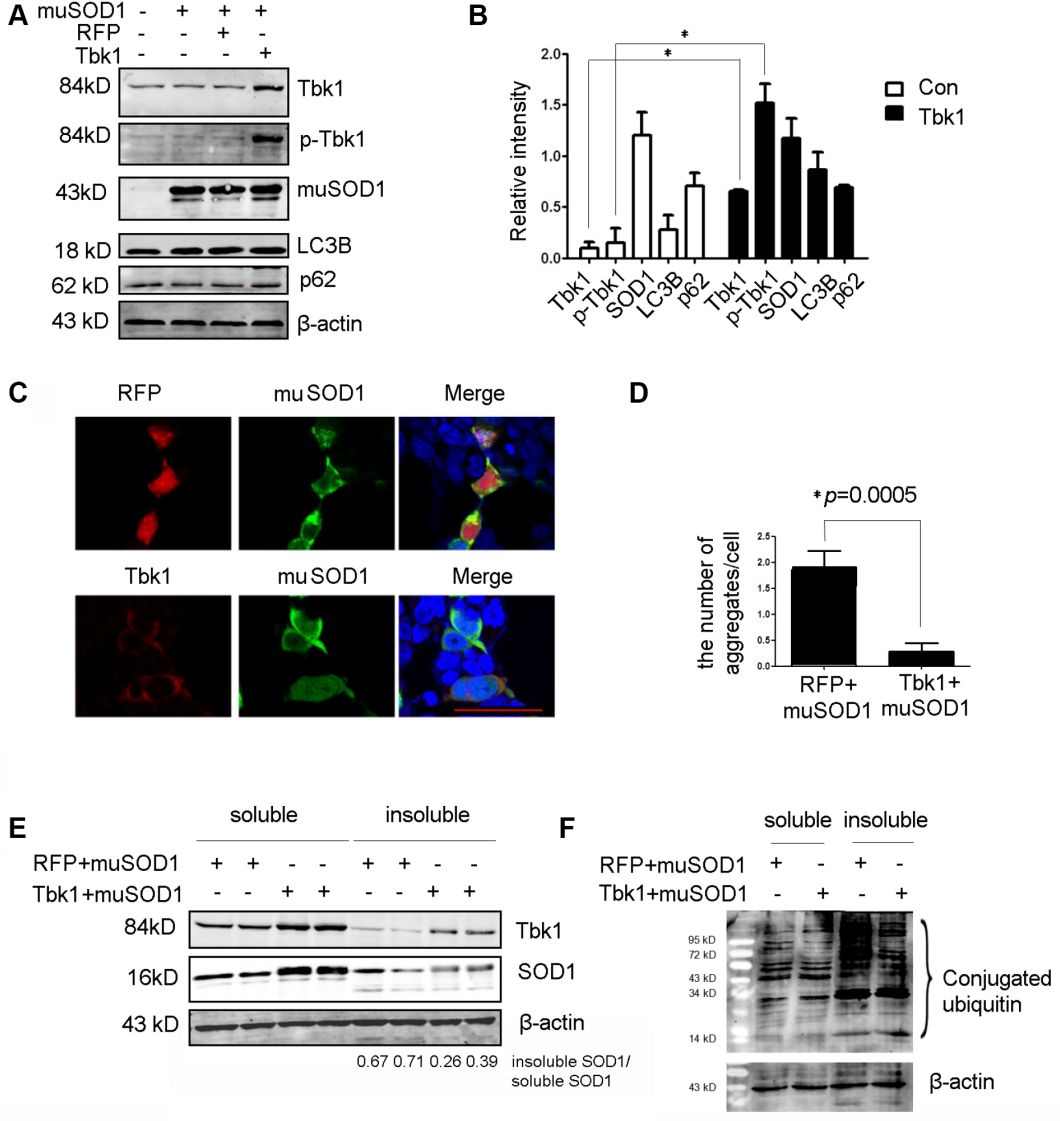
**Over-expression of Tbk1 reduces the number and size of mutant SOD1 aggregates.** (**A**–**B**) Western blot analysis of Tbk1, p-Tbk1, SOD1^G93A^, LC3B, and p62 in NSC-34 cells transfected with mutant SOD1 (mu SOD1; i.e. SOD1^G93A^) and either Tbk1 or a RFP control; representative blot (**A**) and summary graph of densitometric quantification data (**B**) (n = 3); *P < 0.05, compared to control. (**C**–**D**) Analysis of mutant SOD1 aggregates by confocal microscopy in NSC-34 cells co-transfected with mutant SOD1-GFP (mu SOD1-GFP) and Tbk1 or RFP; representative images (**C**) and summary graph (**D**) (n = 10); *P < 0.05, compared to control. (**E**–**F**) Quantification of soluble and insoluble mutant SOD1 fractions by western blot in cells over-expressing Tbk1 (n = 3); *P < 0.05, compared to control.

Conversely, to assess whether Tbk1 over-expression could enhance the degradation of mutant SOD1, NSC34 cells were co-transfected with mutant SOD1^G93A^-encoding plasmids and either a Tbk1 expression vector or a control (RFP) vector. Tbk1 over-expression led to a substantial increase in both Tbk1 and p-Tbk1, but did not alter SOD1^G93A^ expression (Figure 10A, 10B). We also examined the effect of Tbk1 over-expression on autophagy induction by assessing LC3B and p62 levels. No expression changes were observed in these proteins, indicating that increased Tbk1 expression is insufficient to promote autophagy (Figure 10A, 10B). Interestingly, however, Tbk1 over-expression prominently reduced the number and size of SOD1^G93A^ aggregates (Figure 10C–10D). Furthermore, western blot analysis revealed that Tbk1 expression increased soluble SOD1^G93A^ levels while reducing insoluble SOD1^G93A^, causing a 37% reduction in the insoluble/soluble mutant SOD1 ratio compared to control RFP cells (Figure 10E). Meanwhile, Tbk1 over-expression also decreased the level of polyubiquitinated proteins in the insoluble fraction (Figure 10F).

### Tbk1 over-expression extends survival of ALS transgenic mice

Considering that Tbk1 might be involved in the degradation of mutant SOD1, we compared Tbk1 protein levels in mutant SOD1 mice and littermate controls.

**Figure 11:**
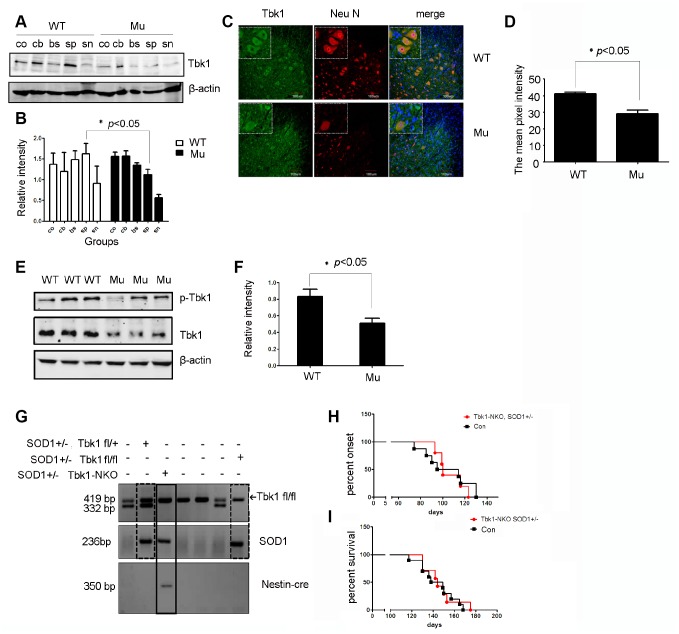
**Analysis of Tbk1 expression in ALS transgenic mice.** (**A**–**B**) Western blot analyses of neural Tbk1 expression in SOD1^G93A^ transgenic mice (co: cortex, cb: cerebellum, br: brain stem, sp: spinal cord, sn: sciatic nerve; n = 3); *P < 0.05, compared to WT littermates. (**C**–**D**) Tkb1 distribution in the spinal cord evaluated by confocal microscopy. Bar = 100 μm. (**E**–**F**) Western blot analysis of phosphorylated Tbk1 in the spinal cord of SOD1^G93A^ mice; representative blot (**E**) and summary graph of densitometric analysis (**F**); (n = 3); *P < 0.05, compared to WT littermates. (**G**) Genotyping of Tbk1-NKO, SOD1G93A^+/-^ mice established by crossing Tbk1-NKO mice with SOD1G93A^+/-^ mice. (**H**–**I**) Disease symptoms onset and survival times recorded for Tbk-NKO, SOD1G93A^+/-^ mice; no differences were observed compared with other genotypes, i.e. Tbk1^fl/fl^, SOD1G93A^+/-^ mice, Tbk1^fl/+^, SOD1G93A^+/-^ mice, and Nestin-cre^+/-^, Tbk1 ^fl/+^, SOD1G93A^+/-^ mice (n = 6-7).

Western blot analysis revealed that the expression of Tbk1 and p-Tbk1 in the spinal cord was profoundly decreased in mutant SOD1^G93A^ mice (Figure 11A, 11B, and 11E, 11F). Furthermore, immunofluorescence revealed that Tbk1 was mainly located in motor neurons (Figure 11C, 11D), and that both the number of the Tbk1-positive cells and the intensity of Tbk1 staining were reduced in mutant SOD1 mice. These results suggest that mutant SOD1 protein is associated with Tbk1 degradation *in vivo*.

It is currently unclear whether Tbk1 deficiency in neuronal cells is sufficient for promoting ALS development. To address this question, we crossed Tbk1-NKO mice with SOD1^G93A^ transgenic mice (Figure 11G). However, to our surprise, neuron-specific Tbk1 deficiency did not significantly affect disease onset or survival in these mice (Figure 11H–11I). These results suggest that Tbk1 deficiency in neuronal cells does not accelerate ALS-FTD symptoms driven by mutant SOD1.

**Figure 12:**
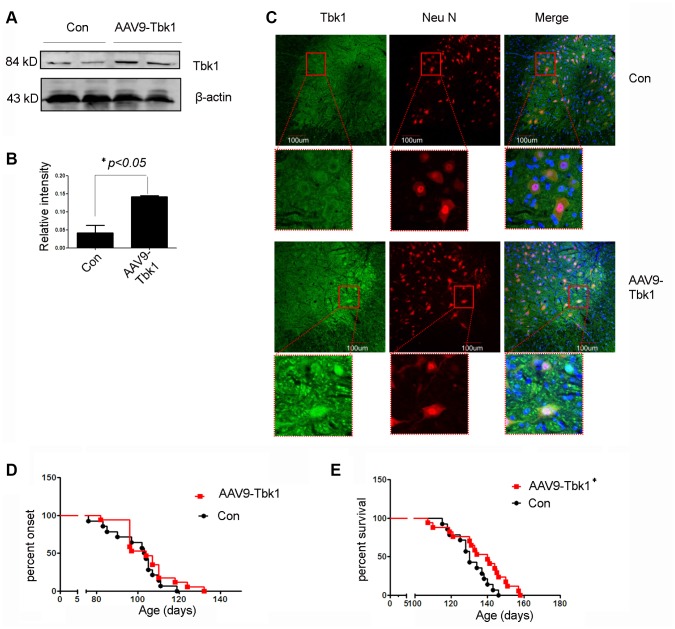
**Tbk1 over-expression prolongs survival of ALS mice.** (**A**–**C**) Tkb1 expression and distribution in the spinal cord of SOD1^G93A^ mice injected (ICV) with an AAV9-Tbk1 vector (n = 3); *P < 0.05, compared to SOD1^G93A^, AAV9-GFP control mice. (**D**) Disease onset and (**E**) survival rate of SOD1^G93A^ mice injected with AAV9-Tbk1 or AAV9-GFP control (Con) (n = 14-17); *P < 0.05, compared to control (viral titer = 1*10^12^ vg/ml).

The decrease in SOD1^G93A^ aggregation induced by Tkb1 over-expression in cultured motor neurons raise the intriguing possibility that Tbk1 might have a protective effect on mutant SOD1-induced ALS. To examine this possibility, we introduced recombinant adeno-associated viruses (AAV) encoding Tbk1 (AAV9-Tbk1) or AAV9-GFP to SOD1^G93A^ transgenic mice by ICV injection. Western blot analyses confirmed increased Tbk1 expression in the spinal cord of mice treated with AAV9-Tbk1 (Figure 12A, 12B). Immunofluorescence staining demonstrated again that Tbk1 was mainly located in motor neurons (Figure 12C), with 43% of ventral horn cells staining positive for Tbk1 and Neu N. Although AAV9-Tbk1 administration did not delay disease onset in SOD1^G93A^ mice [GFP: 105 ± 1.5 days (n = 14), Tbk1: 104 ± 5.7 days (n = 17)], it significantly extended lifespan (140 ± 8 days) compared with mice injected with AAV9-GFP (130 ± 1.8 days) (Figure 12D, 12E). In addition, motor function in AAV9-Tbk1-treated mice was moderately improved (stride length = 2.9 ± 0.7 cm and 3.7 ± 0.19 cm for control and AAV9-Tbk1-treated mice, respectively; n = 4; p > 0.05) (Supplementary Figure 3A, 3B). Furthermore, AAV9-Tbk1 administration delayed weight loss in mutant SOD1 mice by one week (n = 11) (Supplementary Figure 3C).

## DISCUSSION

FTD is a heterogeneous neurodegenerative disease that often coexists with ALS. Most commonly, FTD-ALS cases arise from a mutation in the C9orf72 gene, and up to 30% of ALS patients will also develop FTD. Several genetic alterations, including mutations in the Tbk1 gene, are common to both disorders. Tbk1 loss-of-function mutations have been shown to cause FTD and fALS [5] with reported frequencies of 1.1%, 3.4%, and 4.5% for these mutations in FTD, ALS, and FTD-ALS, respectively [26]. Patients with Tbk1 mutations exhibit most often the behavioral variant, rather than the language variant of FTD, with early memory impairment being prevalent in most cases [27]. Our Tbk1-NKO mice reproduced the main symptoms of FTD-ALS, i.e. memory deficits and reduced locomotor activity at advanced age (14 months). Interestingly, however, deletion of Tbk1 in SOD1^G93A^ transgenic mice (a typical model of motor neuron disease/ALS), did not affect symptoms onset nor survival. This may reflect a strong effect of mutant SOD1 that masks any actual contribution of Tbk1 deficiency to disease. Interestingly, Tbk1-over expression significantly reduced protein aggregates and extended the lifespan of SOD1^G93A^ mice. Therefore, restoring Tbk1 function might provide therapeutic benefits for both Tbk1- and SOD1-related neurodegenerative diseases.

Accumulating data demonstrate that Tbk1 critically contributes to clearance of protein aggregates through the autophagy pathway [28]. In addition to Tbk1, mutations in p62 and OPTN genes have been associated with ALS in patient studies [29–30]. It is generally believed that the autophagy regulatory functions of p62 and OPTN require their phosphorylation by Tbk1, since phosphorylated p62 and OPTN efficiently recognize ubiquitinated cargos to facilitate autophagosome formation [22, 31]. We found that ubiquitin and p62 positive aggregates were present in the CNS -especially in the cerebellum- of Tbk1-NKO mice, suggesting impaired autophagic flux.

Clinical research demonstrated mesencephalic and cerebellar atrophy in patients with a p.Glu643del Tbk1 mutation [32]. The cerebellum reciprocally connects with the prefrontal and parietal cortices, which are essential for many aspects of cognition [33]. Cumulative evidence has indicated that the cerebellum participates in various aspects of cognition, such as error-based learning by which changes in behavior are driven by errors. The error is reduced in a continuous manner from trial to trial, continuing until the performance is error free [34–35]. Consistent with cerebellar dysfunction evidenced through classical and immunostaining techniques, our water maze experiments showed that error-based learning was progressively impaired in Tbk1-NKO mice.

In summary, our findings describe structural and biochemical changes in CNS neurons induced by Tkb1 silencing that may underlie those occurring in clinical cases of FTD-ALS. Given the varied and complex functions of Tkb1 and its regulatory role over the autophagy pathway, further research is warranted to define its potential therapeutic value for these devastating diseases.

## MATERIALS AND METHODS

### Animal models

Tbk1^fl/fl^ mice were generated by Taconic and have been described previously [10]. Nestin-cre mice and SOD1^G93A^ transgenic mice (B6SJL-TgN [SOD1-G93A] 1Gur) were obtained from Jackson Laboratories. Tbk1^fl/fl^ mice were crossed with Nestin-cre mice to generate Tbk1^fl/fl^ Nestin-cre (termed neuronal cell-conditional Tbk1 KO or Tbk1-NKO) and Tbk1^+/+^Nestin-cre wildtype (WT) mice. These mice were further crossed with SOD1^G93A^ transgenic mice to produce Tbk1-NKO, SOD1G93A^+/-^ mice. Animals were bred and maintained under controlled conditions (12-h light/dark cycles, 60% ± 10% relative humidity, 22 ± 1°C). Copy number of the hSOD1 gene was evaluated by real-time PCR. Body weight was measured every 7 days starting around 60 days of age.

Animal experiments were carried out according to the laboratory animal management regulations promulgated by the Ministry of Science and Technology of the People’s Republic of China, which are in accordance with the NIH Guide for the Care and Use of Laboratory Animals.

### AAV9-Tbk1 cloning and packaging

AAV-Tbk1 was packaged into pseudotyped AAV9 vectors by transfection of HEK 293T cells. After purification and dialysis, the viral particles were stored at –80 °C. A PCR assay was used to test the vector titer.

### Viral vector delivery

### *Intracerebroventricular injection*

The injection point was identified at 2/5 of the distance from the lambda suture to each eye, approximately 0.8–1 mm lateral from the sagittal suture, halfway between lambda and bregma. Four μl of diluted AAV with 0.05% trypan blue were injected to a depth of approximately 3 mm. The needle was retained for 1 min, and then slowly withdrawn.

### Cell culture and transfection

The NSC-34 cell line was routinely maintained in DMEM (Invitrogen, CA, USA; Cat. No: 21063-029) with 10% heat-inactivated FBS (Invitrogen; Cat. No: 16000-044) and antibiotics (100 IU/mL penicillin and 100 µg/mL streptomycin).

NSC-34 cells were transfected with empty pCI-neo vectors or vectors cloned with SOD1^G93A^ or wild type SOD1 using LipofectamineTM 2000 transfection reagent (Invitrogen; Cat.No:11668-019) following the manufacturer’s protocol. Cells were maintained at 37^o^C in a 5% CO_2_ humidified atmosphere in 25 cm^2^ flasks, with media changes every 2–3 days. NSC34 cells were transfected with SOD1-GFP vector for 48 hours and harvested after digestion with trypsin (0.25% w/v).

### Inhibitors

The proteasome inhibitor MG132 (Cat. No: s1748) was from Beyotime, China. The autophagy inhibitor Bafilomycin A (Cat. No: 196000) was from Sigma, USA.

### Immunohistochemistry

Following transcardiac perfusion with 4% paraformaldehyde, mice brains were removed and further fixed for 24 h in the same fixative. The cerebrum was cut into 20 μm free-floating sections using a Leica VT1000S vibratome. The sections were permeabilized with 0.3% Triton X-100 and then washed three times in 0.01M phosphate-buffered saline (PBS). After blocking with 3% H_2_O_2_ in methanol for 15 min and in 10% horse serum for 1 h at room temperature, the sections were incubated overnight at 4°C with antibodies against Tbk1 (1:400, abcam, ab40676), MAP2 (1:500, Proteintech, 17490-1), NF-M (1:1000, abcam, ab7794), IBA1 (1:500, Wako, 019-19741), p62 (1:500, Sigma, P0067), TDP-43 (1:200, Proteintech, 10782-2-AP), ubiquitin (1:100, Proteintech, 10201-2-AP), VGAT (1:100, SYSY, 131011), or GFAP (1:200, Millipore, MAB360). The sections were subsequently incubated at room temperature with a biotin-conjugated secondary antibody (ZSGB-BIO, 1:200) for 2h, followed by incubation with HRP-conjugated streptavidin (ZSGB-BIO,1:200) for 1h, and 0.03% diaminobenzidine as a chromogen for 10 min. Slides were mounted and analyzed by light microscopy (Olympus BX51).

### Confocal laser scanning microscopy

Brain sections (obtained as described above) were washed three times in 0.3% Triton X-100/PBS and blocked in 10% horse serum for 30 min. Primary antibodies against Tbk1 (1:400, abcam, ab40676), phospho-Tbk1 (1:100, Cell signaling, 5483), Neu N (1:500, Millipore, MAB377), GFAP (1:200, Millipore, MAB360), p62 (1:500, Sigma, P0067), or MAP2 (1:500, Proteintech, 17490-1) were applied overnight at 4°C. After washing, the sections were incubated with fluorescent secondary antibodies for 1 h at room temperature. The slides were observed using a fluorescence confocal microscopy (Olympus FV1000).

### Western blotting

Protein expression in the cortex and cerebellum was quantified by western blotting. Total protein was extracted using a total protein extraction kit (Applygen Technologies Inc., P1250). Fifty micrograms of protein from each sample were separated on 10% or 12% SDS–PAGE gels and transferred onto PVDF membranes. The membranes were incubated overnight at 4°C with primary antibodies, including β-actin (1:2000, Proteintech, 60008-1-Ig), Tbk1 (1:1000, abcam, ab40676), phospho-Tbk1 antibody (1:1000, Cell signaling, 5483), p62 (1:1000, Sigma, P0067), phospho-p62 (Phospho-Ser403) (1:1000, SAB, 12804), LC3B (1:500, Sigma, L7543), TDP-43 (1:1000, Proteintech, 10782-2-AP), GFAP (1:1000, Millipore, MAB360), ubiquitin (1:1000, Proteintech, 10201-2-AP), or hSOD1 (1:10000, Abcam, ab51254). Following incubation with fluorescent secondary antibodies for 1 h at room temperature, an Odyssey Infrared Imaging System (LI-COR, Lincoln, NE) was used to detect the bands of interest.

### Electron Microscopy

Tissues were fixed in 4% glutaraldehyde and treated with 1% OsO_4_ in 0.1 M PBS. The samples were dehydrated through graded acetones and embedded in EPON 812. Ultrathin sections (70 nm) were placed on a copper grid and stained with uranyl acetate and lead citrate. The samples were observed by transmission electron microscopy (TEM, JEM-1230). Synapses were identified based on the following features: parallel pre-and post-synaptic membranes, a discernable synaptic cleft, a postsynaptic density, and synaptic vesicles. Synapse counts were obtained from 6-7 TEM images from each animal.

### Golgi and Bielschowsky stains

Dendritic spines were studied using Golgi-Cox staining (Hito Golgi-Cox OptimStain TM PreKit Hitobiotec Corp.) according to the manufacturer’s instructions. Briefly, the animals were sacrificed and their brains immediately removed and rinsed in 0.1M phosphate buffer. Brains were immersed in a Golgi-Cox solution, which was renewed after 12 h, and then stored at room temperature in the dark for 12 days. Brains were then transferred to Solution 3 and stored at 4°C for 48 h in the dark before inclusion in low gelling temperature agarose and sectioning (200 μm thickness). The sections were next transferred onto gelatin-coated slides, air-dried at room temperature in the dark, rinsed with distilled water, stained in a developing solution, dehydrated, cleared, and coverslipped. Five to nine microscopic fields were selected and quantified using Image J software. Spine density was determined as the number of spines per 20 μm of dendrite length.

Staining of nerve fibers was carried out using the Hito Bielschowsky OptimStain Kit (HTKNS1126). Briefly, the slides were incubated with Solution-1 for 15 min, followed by immersion in Developer solution for 1–8 min until tissues became golden-brown. After incubation with Solution 4, sections were rinsed with distilled water, dehydrated, cleared, and coverslipped.

### Behavioral tests

### *Spatial learning test*

Morris Water Maze task procedures were based on previous descriptions with some modifications [32]. The mice’s ability to reach a visible platform in the Morris water maze was first assessed to rule out any sensory-motor-related impairment. Then, 4 trials per day were conducted over 5 days. Each trial ended either when an animal climbed onto the platform or when a maximum of 60 s elapsed. At day 6, all mice were given a probe test for 60 s with the platform removed from the pool. These tests were conducted on 5 months old and 14 months old mice. Data collection and analysis were performed using a digital tracking device.

### Motor function test

A rotarod (Rota-Rod; Ugo Basile, Gemonio, Italy) was used to assess motor function in mice. With arbitrary cut-off time of 180s, three once-a-week trials were performed, and the longest time was recorded. Onset of disease was determined as a reduction in rotarod performance between weekly time points.

### *Accelerated rotarod testing*

Mice were placed on the top of the revolving beam for 4 successive trials/day for 2 days, with 20-min intertrial intervals. The rod was accelerated gradually from 4 to 28 rpm over 2 min. Latencies before falling from the rod were recorded. Footprint measurements were recorded during continuous locomotion.

Stride length was measured as the distance between prints and the average of three stride lengths was calculated. Stretch width was recorded in mice suspended by the tail.

Forelimb grip strength was measured using a Chatillon (Largo, FL,USA) DFIS-10 digital force gauge. Mice were allowed to grab the wire screen attached to the instrument and then gently pulled back horizontally from the wire by the tail. Grip strength represents the maximum force recorded prior to release of the mouse’s paws from the wire bars.

### Statistical analysis

Data were analyzed using SPSS 13.0 software. Lifespan was compared between groups of mice by the Kaplan–Meier method. Comparisons among multiple groups were performed using one-way ANOVA followed by Student–Newman–Keuls or Dunn’s T tests. All values are expressed as the mean ± S.E, and P < 0.05 was set as statistically significant.

## Supplementary Material

Supplementary Figures
