# Supplementary material for: Deletion of Tbk1 disrupts autophagy and reproduces behavioral and locomotor symptoms of FTD-ALS in mice
Source: Aging (Albany NY). 2019 Apr 30;11(8):2457–76. doi: 10.18632/aging.101936 (PMC6519994; doi:10.18632/aging.101936)
Supplement: Supplementary Figures [file aging-11-101936-s001.pdf]

SUPPLEMENTARY FIGURES

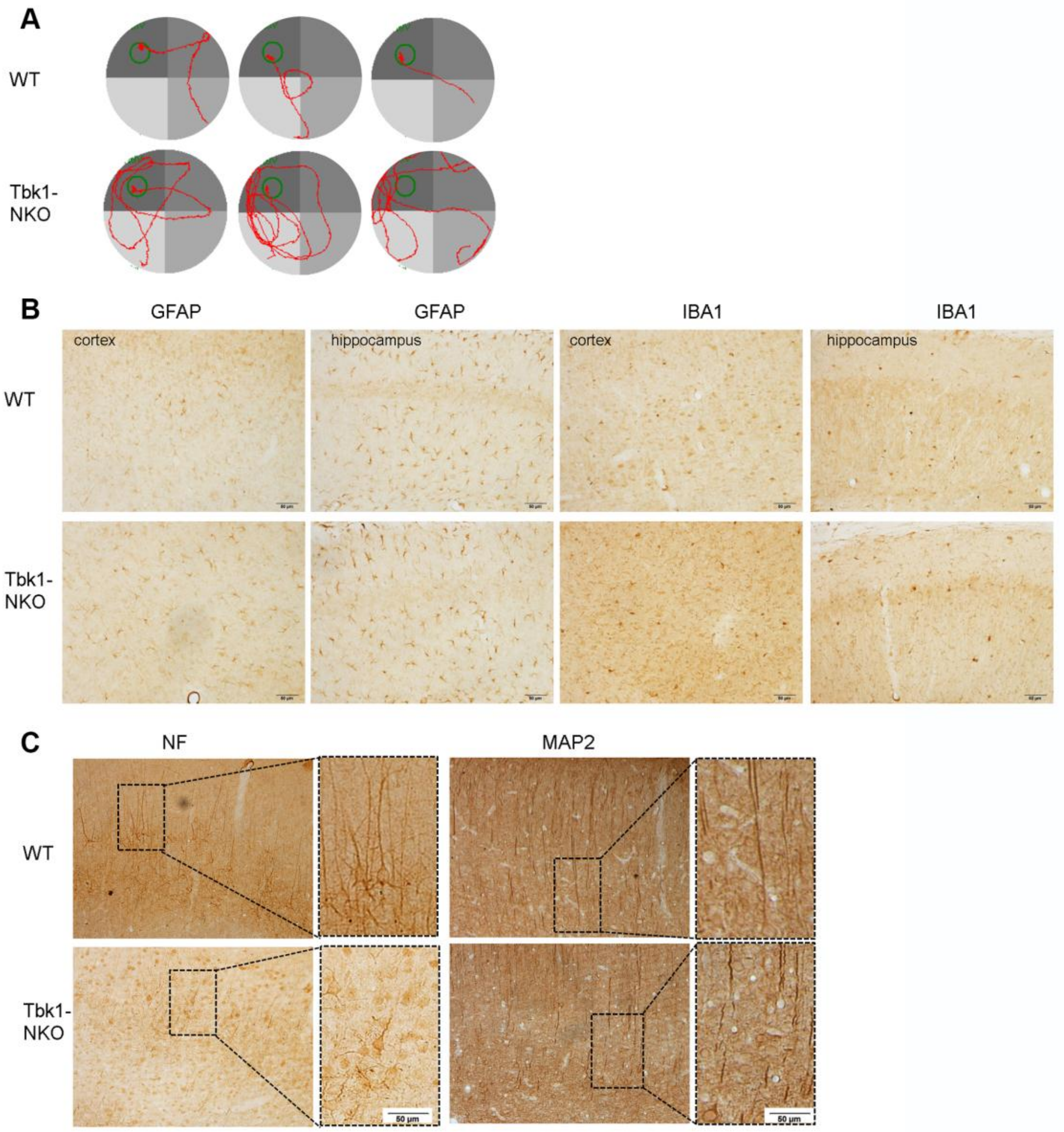

Supplementary Figure 1. (**A**) Representative swimming path of Tbk1-NKO and WT mice in the Morris water maze experiment. (**B**–**C**) GFAP, IBA1 (n = 3; bar = 20 μm), Neurofilament, and MAP2 immunostaining of brain sections from WT and Tbk1-NKO mice (n = 3; bar = 50 μm).

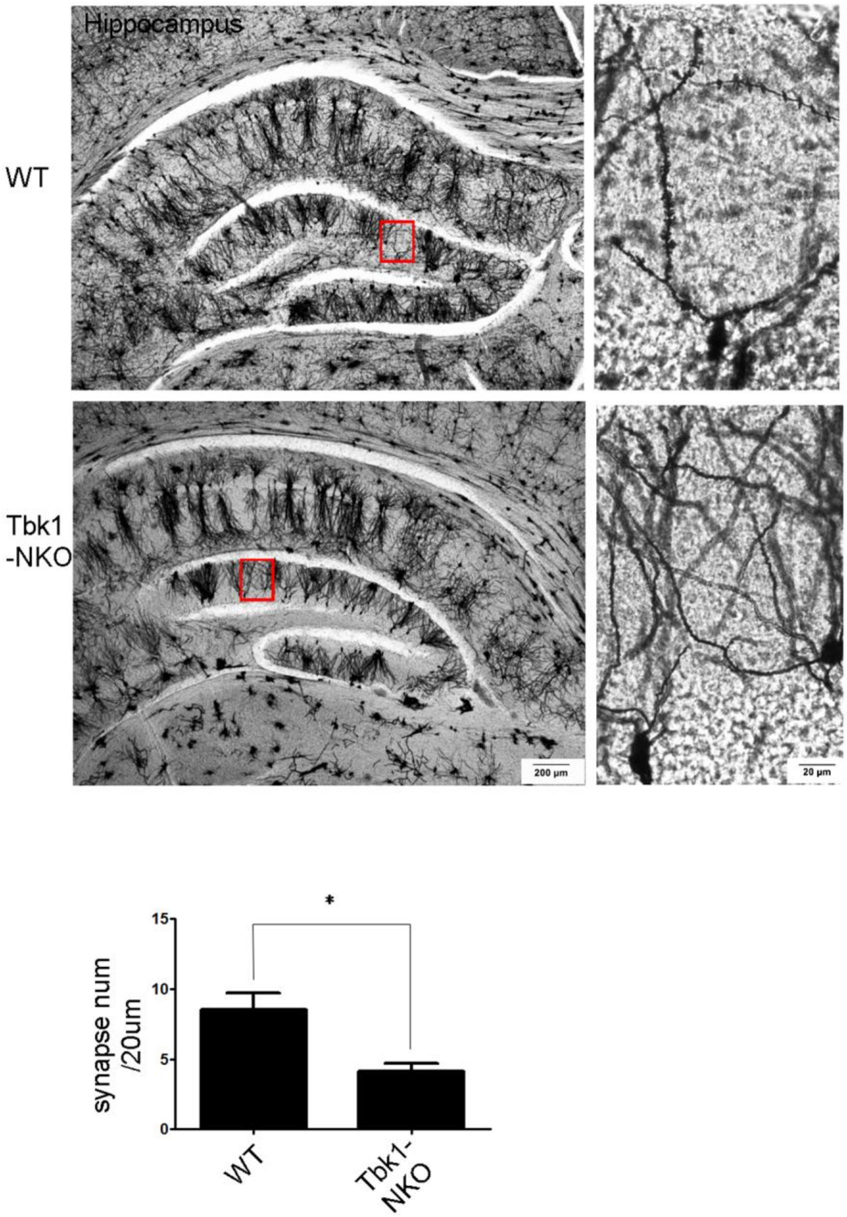

Supplementary Figure 2. Golgi-Cox staining of the hippocampus of Tbk1-NKO and WT mice (n = 3). Dendritic spine density was measured using Image J (n = 5); *P < 0.05, compared to WT mice.

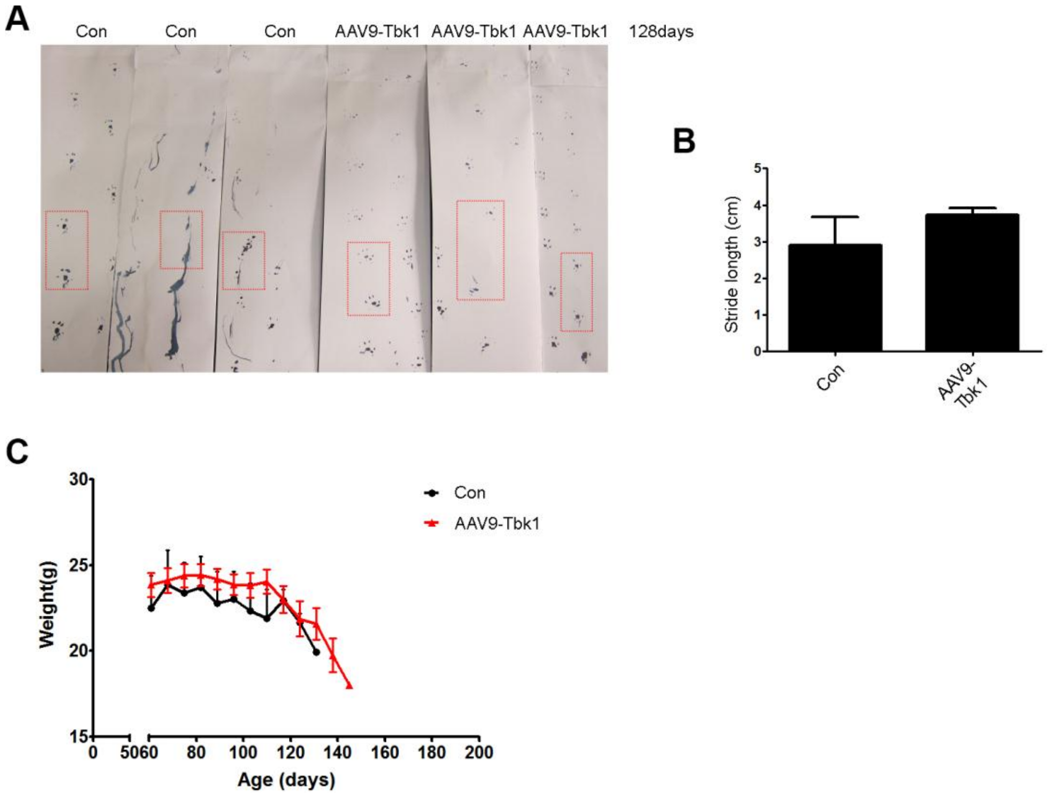

Supplementary Figure 3. (A–B) Mice footprints and (C) body weights were evaluated in mutant SOD1 mice after ICV injection of AAV9-Tbk1 and AAV9-GFP (Con) vectors.
